# Supplementary material for: Whole-Transcriptome RNA Sequencing Reveals the Global Molecular Responses and CeRNA Regulatory Network of mRNAs, lncRNAs, miRNAs and circRNAs in Response to Salt Stress in Sugar Beet (Beta vulgaris)
Source: Int J Mol Sci. 2020 Dec 30;22(1):289. doi: 10.3390/ijms22010289 (PMC7795855; doi:10.3390/ijms22010289)
Supplement: Supplementary file 1 [file ijms-22-00289-s001.zip › Supplementary File/Supplementary Fig S1~3.pdf]

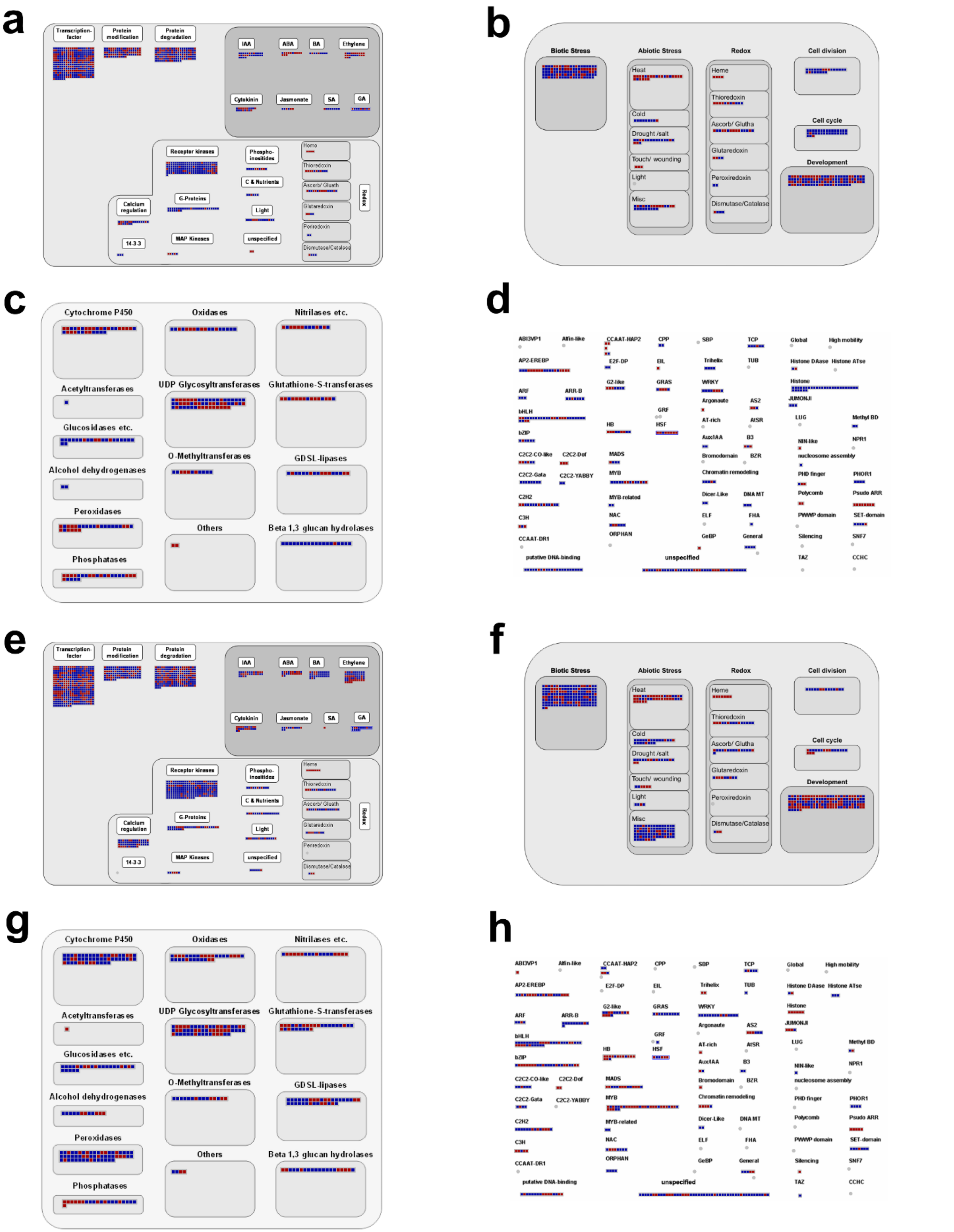

Fig. S1 Mapman analysis of DEmRNAs in leaves (a, b, c, d) and roots (e, f, g, h) under salt stress. DEmRNAs mapping to Regulation_overview in leaves (a) and roots (e); DEmRNAs mapping to Cellular_response_overview in leaves (b) and roots (f); DEmRNAs mapping to Large_enzyme_families in leaves (c) and roots (g); DEmRNAs mapping to transcription in leaves (d) and roots (h). Each square represents a DEmRNA mapping to a specific metabolic pathway, with red being up-regulated and blue being down-regulated.

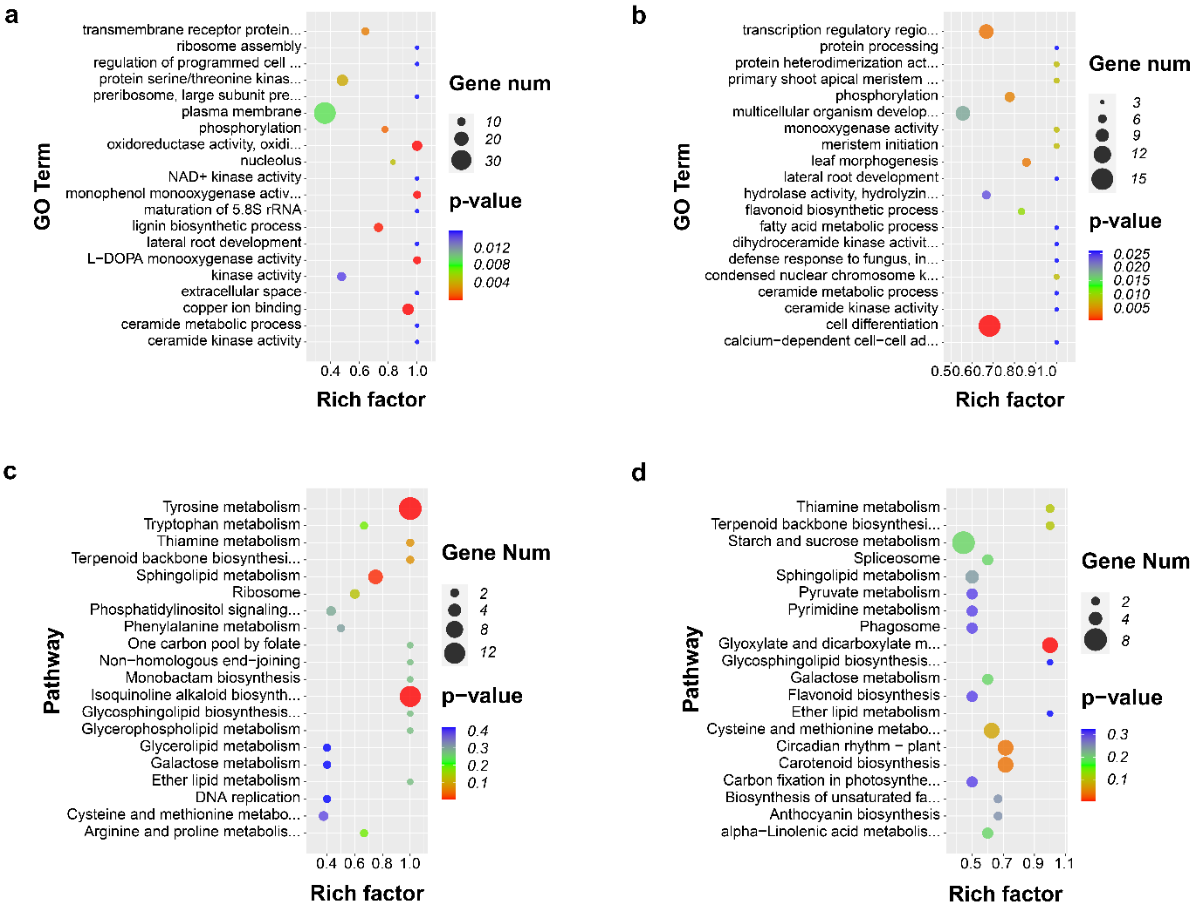

Fig. S2. GO and KEGG enrichment analysis of DEmRNAs targets. (a) GO enrichment analysis of targets of DEmiRNAs in leaves; (b) GO enrichment analysis of targets of DEmiRNAs in roots; (c) KEGG enrichment analysis of targets of DEmiRNAs in leaves; (d) KEGG enrichment analysis of targets of DEmiRNAs in roots.

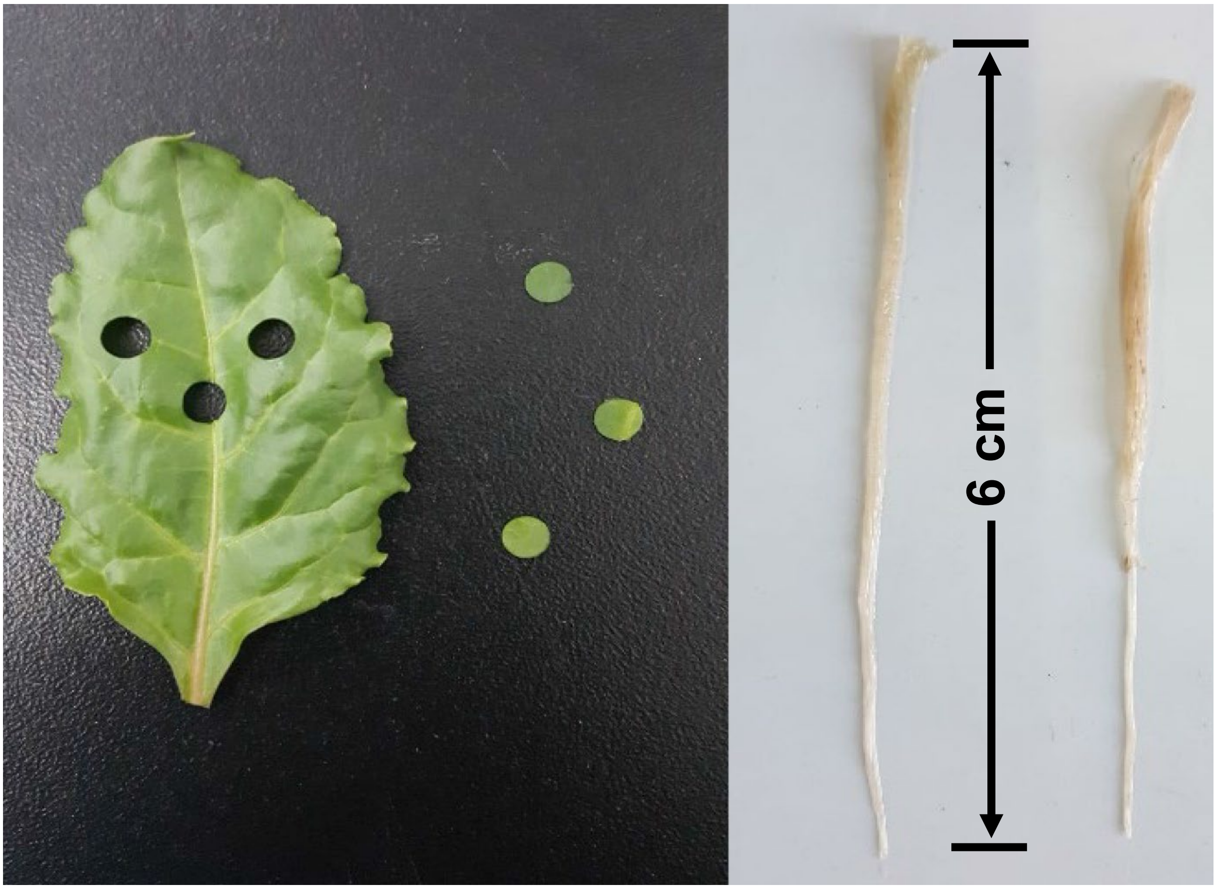

Fig. S3. Sampling location. Left: the third-pair of euphylla from six plants were taken and mixed for leaf sequencing. Right: a length of 6 cm from the root tip was collected from six plant samples and mixed for root sequencing.
